# The efficacy of intraoperatie continuous glucose monitoring in patients undergoing liver transplantation: a study protocol for a prospective randomized controlled superiority trial

**DOI:** 10.1186/s13063-023-07073-x

**Published:** 2023-02-01

**Authors:** Yi Duan, Zuo-Zhi Li, Pan Liu, Lei Cui, Zhifeng Gao, Huan Zhang

**Affiliations:** 1 Department of Anesthesiology, Beijing Tsinghua Changgung Hospital, School of Clinical Medicine, Tsinghua University, No. 168 Litang Road, Beijing, 102218 China; 2 Department of Special Care Center, Fuwai Hospital, National Center for Cardiovascular Diseases, Chinese Academy of Medical Sciences and Peking Union Medical College, Beijing, 100037 China

**Keywords:** Continuous glucose monitoring, Coefficient of variation, Variability, Liver transplantation, Prognosis

## Abstract

**Background:**

The high incidence of intraoperative glucose dysregulations in liver transplantation (LT) is related to the lack of highly orchestrated control of intraoperative blood glucose. Glucose monitoring based on a single arterial blood gas test can only provide a simple glucose profile and is insufficient in monitoring intraoperative glycemic variability (GV), which is not conducive to controlling GV and may have a lag in the management of hyper/hypoglycemia. Continuous glucose monitor (CGM), which has been successfully applied in the management of chronic disease in diabetes, provides more detailed blood glucose records and reflect GV parameters such as coefficient of variation (CV%). However, its effectiveness and accuracy for guiding blood glucose management in major surgeries remains unclear.

**Methods:**

This is a single-center, randomized, controlled, superiority trial. One hundred and eighty patients scheduled for orthotopic LT will be recruited and randomized into two groups. All patients are monitored for intraoperative glucose using CGM combined with arterial blood gas (ABG). In the intervention group (group CG), ABG will be performed when CGM value is < 6.1 mmol/L or > 10.0 mmol/L, or the rate of change of CGM value > 1.67 mmol/(L·min). In the control group (group G), intraoperative ABG tests will be performed every 2 h, and the frequency of ABG tests will be adjusted based on the previous arterial glucose result. Patients in both groups will have their blood glucose adjusted according to arterial glucose values and a uniform protocol. Surgical and other anesthetic management is completed according to standard LT practices.

**Discussion:**

This study intends to investigate the effectiveness of CGM-based intraoperative glucose management and its impact on the prognosis of LT patients by comparing the GV, mean glucose values, and the incidence of hypo/hypoglycemic events guided by the above two glucose monitoring methods.

**Trial registration:**

This study is registered at www.chictr.org.cn on January 4, 2022, under the registration number ChiCTR2200055236.

## Administrative information

**Table:** 

Title {1}	The efficacy of intraoperative continuous glucose monitoring in patients undergoing liver transplantation: a study protocol for a prospective randomized controlled superiority trial
Trial registration {2a and 2b}	This study is registered at www.chictr.org.cn on January 4, 2022, under the registration number ChiCTR2200055236
Protocol version {3}	V2.0 2021.08.29
Funding {4}	Youth Start-up Fund of Beijing Tsinghua Changgung Hospital (No.12021C1005)
Author details {5a}	Yi Duan^1^, Zuo-Zhi Li^2^, Pan Liu^1^, Lei Cui^1^, Zhifeng Gao^1^, Huan Zhang^1^ 1 Department of Anesthesiology, Beijing Tsinghua Changgung Hospital, School of Clinical Medicine, Tsinghua University, No. 168 Litang Road, Beijing, 102218, China 2 Department of Special Care Center, Fuwai Hospital, National Center for Cardiovascular Diseases, Chinese Academy of Medical Sciences and Peking Union Medical College, Beijing, 100037, China
Name and contact information for the trial sponsor {5b}	Beijing Tsinghua Changgung Hospital
Role of sponsor {5c}	Provide research funding, academic guidance, and research supervision

## Introduction

### Background and rationale {6a}

Maintaining blood glucose levels within a reasonable range is a key target of perioperative blood glucose management. Similar to the glucose control performed in regular diabetic patients, strict perioperative glucose control reduces risk of postoperative hyperglycemia-related adverse events such as cardiovascular events [1], short-term mortality [2], and microvascular complications [3] and reduces the risk of surgical site infections (SSIs) and secondary hospitalization rates [4–9]. However, unlike chronic diabetes management, intraoperative glycemic changes are more complex and dramatic due to various exogenous factors, such as fasting, fluid infusion, and traumatic stimuli [3, 10], which makes intraoperative precise glycemic management more difficult. Recent studies demonstrated that hyperglycemia is one of the common complications in liver transplant recipients in the perioperative period, with an incidence ranging from 6.7 to 94% [11, 12], and 45.9% of recipients develop severe hyperglycemia (> 13.4 mmol/L) in the neohepatic phase [13]. In our previous retrospective study, we observed that the incidence of intraoperative glucose peaks between 7.8 and 13.4 mmol/L during liver transplantation (LT) was 68.6%, and the incidence of peak glucose > 13.4 mmol/L was 24.8%. The high incidence of intraoperative glucose dysregulation suggests an urgent need for orchestrated blood glucose control in these patients.

Recent diabetes management guidelines suggest that a sound glycemic management strategy should focus on three pillars: persistent hyperglycemia, hypoglycemia, and glycemic variability (GV) [14–16]. Previous studies have shown that glucose management measures based on intermittent arterial blood gas monitoring of blood glucose have not adequately reduced hyperglycemia-related complications [17], possibly attributable to (1) poor control of intraoperative hyperglycemia [18–20] and (2) ineffective control of intraoperative GV [21, 22]. GV, defined as the degree of fluctuation of blood glucose levels between peak and trough values, serves as a novel prognostic feature of glucose management that is thought to further reduce the incidence of diabetes-related complications [16]. Depending on the observation time frame, measures of GV can generally be divided into two categories: long-term and short-term [15, 23, 24]. Among them, the coefficient of variation (CV%) is considered to be the most appropriate indicator for assessing intraday GV, and it is also easy to perform and interpret when evaluating intraoperative glucose fluctuations. Higher CV% values indicate worse short-term glycemic control, and CV% > 36% is associated with increased risk of hypoglycemia and/or mortality [25, 26]. Rangasamy et al. [27] consolidated the prognostic values of CV%, as a short-term assessment of GV, in predicting serious adverse events after cardiac surgery (OR = 1.91). Consistently, we recently reported that the incidence of early postoperative graft dysfunction was significantly higher in recipients with intraoperative CV% higher than 28% in LT than in recipients with low CV% (10.8% vs 0%, *P* < 0.01). However, conventional glucose monitoring based on a single arterial blood gas test is time-consuming and labor-intensive, and the measured results represent only the instantaneous concentration at the time of blood collection, thus providing only a simple glucose profile [28]. Therefore, there is an urgent need for viable means of intraoperative GV monitoring and prevention of excessive GV [29].

Continuous glucose monitor (CGM) is a wearable device that automatically detects glucose levels from interstitial fluid or blood every 5 min [30, 31]. Among them, interstitial fluid CGM has been widely used in the chronic disease management of diabetic patients due to the advantages of minimally invasive, stability and less complications [32], and its related products have been approved and marketed by the National Medical Products Administration (NMA) of China. Compared with blood gas examination, CGM provides more detailed blood glucose readings. In addition, CGM evaluates GV parameters such as CV% and has gradually become the gold standard for assessing short-term blood GV in recent years [33, 34]. Given that glucose changes are more drastic under intraoperative stress and there is a different degree of lag in glucose concentration changes in interstitial fluid than in blood, whether CGM based on interstitial fluid can accurately and effectively guide intraoperative glucose management still needs to be validated by high-quality prospective studies.

### Objectives {7}

Therefore, we propose the hypothesis that interstitial fluid-based CGM for intraoperative adjunctive glucose monitoring reduces the coefficient of variation of blood glucose and improves glucose control, which may help improve the prognosis of patients after liver transplantation.

### Trial design {8}

**Fig. 1:**
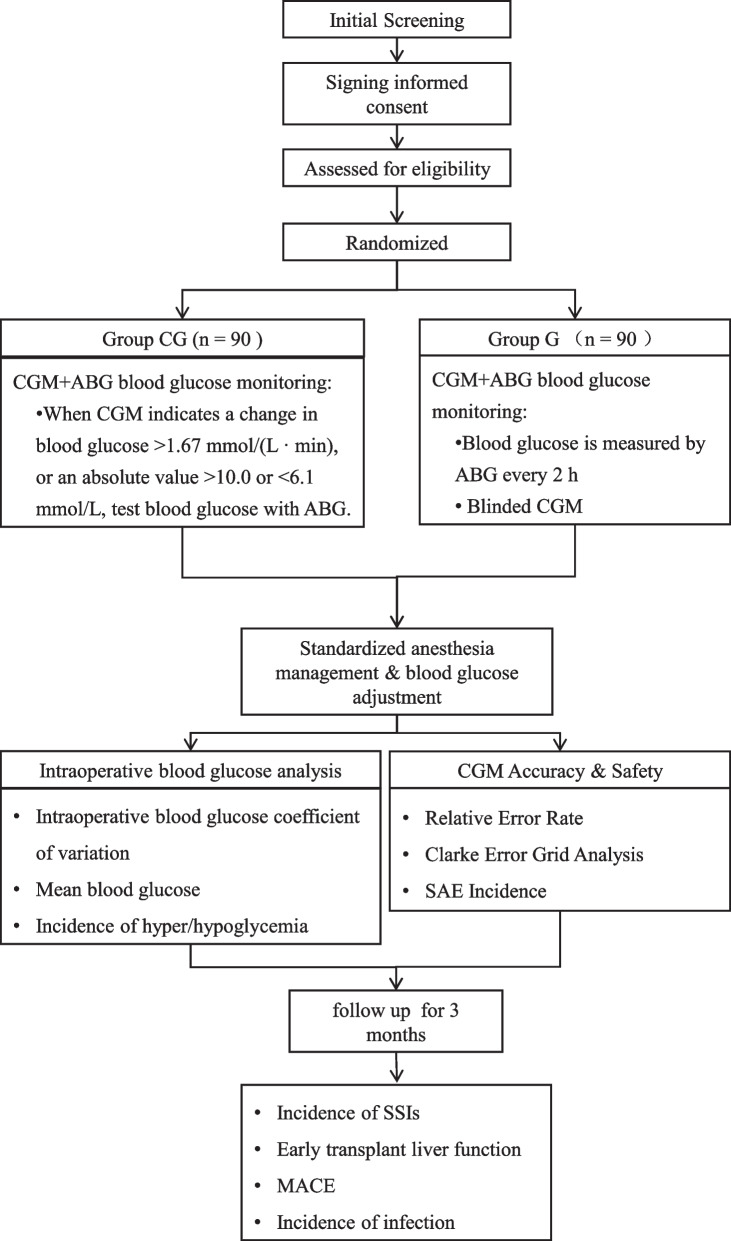
Study design

To address the shortcomings of the current glucose monitoring, which lacks continuity and dynamics, and cannot reflect the trend of glucose changes, this study explores the feasibility and effectiveness of continuous glucose monitoring (CGM) in major surgery such as liver transplantation using a prospective randomized, controlled, superiority clinical trial [35]. Furthermore, we will compare the effects of CGM-based and traditional blood glucose management measures on the reduction of intraoperative blood glucose variability in liver transplantation and the improvement of postoperative outcomes (e.g., surgical site infection). The study design is shown in Fig. 1.

## Methods: participants, interventions, and outcomes

### Study setting {9}

This is a single-center, randomized, controlled study, which will be conducted at Beijing Tsinghua Changgung Hospital, China. The study intends to enroll 180 patients who are scheduled for allogeneic orthotopic liver transplantation.

All patients who met the inclusion and exclusion criteria were randomly assigned to the intervention and control groups. Intraoperative glucose monitoring will be performed by CGM combined with arterial blood gas (ABG) in the intervention group (CG group) and by ABG alone in the control group (G group). The effectiveness of CGM- and ABG-based intraoperative glucose management and their impact on the prognosis of LT recipients will be evaluated using GV, mean glucose values, and the incidence of hyperglycemic or hypoglycemic events. The study, approved by the Ethics Committee of Beijing Tsinghua Changgung Hospital (approval number: 21288–0-02), will follow the Declaration of Helsinki (2013 version). The study is registered at www.chictr.org.cn (ChiCTR2200055236).

### Eligibility criteria {10}

#### Inclusion criteria

1. Age 18 ~ 65 years
2. Type 2 diabetes or preoperative glycosylated hemoglobin (HbA1C) > 6.5%
3. Scheduled for allogeneic orthotopic liver transplantation
4. ASA class II ~ III
5. Signed written informed consent

#### Exclusion criteria

1. Diabetes mellitus of any type other than type 2
2. Model for end-stage liver disease (MELD) score ≥ 30
3. Severe preoperative infection
4. Severe preoperative cardiovascular instability, including unstable angina pectoris, left ventricular ejection fraction < 30%, and malignant arrhythmia.
5. Patient refusal

#### Criteria for removal from the trial

1. Failure to manage anesthesia or blood glucose in accordance with the study protocol
2. Intraoperative massive blood loss, defined as blood loss exceeding 50% of systemic blood volume within 3 h or blood loss at a rate of > 150 ml/h
3. Death within 3 months after LT
4. Loss of follow-up
5. Voluntary patient withdrawal from the study
6. Failure to record study data

### Who will take informed consent? {26a}

The investigator (Lei Cui) will initially screen potential participants based on the surgical schedule and e-case the day before the surgery, then presents the details of the purpose, content, possible benefits, and risks of this study in a separate, undisturbed room, and obtains a signed informed consent.

### Additional consent provisions for collection and use of participant data and biological specimens {26b}

N/A. This trial does not involve collecting biological specimens for storage.

### Interventions

#### Explanation for the choice of comparators {6b}

In this study, blood glucose in the control group will be measured by ABG testing at 2-h intervals, and the monitoring frequency will be adjusted according to the single glucose value, which is the most common way of blood glucose monitoring at present. However, this monitoring method has the disadvantages of not reflecting GV and easily missing the diagnosis of blood glucose disorder. In the intervention group, CGM combined with ABG test will be used for blood glucose monitoring. In brief, ABG test will be performed to verify the blood glucose value when the CGM monitoring value is abnormal (< 7.8 mmol/L or > 10 mmol/L). The blood glucose intervention in both groups will be based on the blood glucose values obtained from ABG test.

### Intervention description {11a}

#### Blood glucose management

1. Blood glucose monitoring

Intraoperative glucose monitoring is performed in both groups using CGM (Guardian RealTime, Medtronic, USA) in combination with arterial blood gas. A disposable glucose monitoring probe will be placed in the patient's upper arm by the investigator 6 h before surgery and connected to the monitoring system.

In the CG group, intraoperative ABG testing will be performed if the CGM value is < 6.1 mmol/L or > 10.0 mmol/L, or the rate of change of CGM value > 1.67 mmol/(L·min). Blood glucose adjustment will be performed according to the arterial blood glucose value.

In the G group, CGM monitoring results were blinded to patients and investigators. Intraoperative arterial blood gas checks will be performed every 2 h, and blood glucose adjustments will be made based on blood glucose results, and the frequency of blood gas tests will be adjusted as needed. CGM is used to record intraoperative glucose data only.

2) Blood glucose management target: The intraoperative blood glucose management target is 7.8 ~ 10 mmol/L in both groups.
3) Blood glucose-lowering measures: see Table 1 for the intraoperative glucose management measures for patients in both groups.

**Table 1:** Blood glucose intervention

Perioperative target blood glucose: 7.8 ~ 10 mmol/L • If ABG-derived blood glucose > 10 mmol/L measured for the first time, start insulin infusion Insulin dose and mode of administration: • Loading dose: 0.1 U/kg, I.V Maintenance dose: (blood glucose value/5)U/h, infusion			
**Blood glucose (mmol/L)**	**If BG increased from previous measurement**	**BG decreased from prior measurement by ≤ 1.67 mmol/L**	**BG decreased from prior measurement by > 1.67 mmol/L**
**> 13.4**	Increase rate by 3U/h	Increase rate by 3U/h	No change in rate
**11.7 ~ 13.4**	Increase rate by 2U/h	Increase rate by 2U/h	No change in rate
**10.0 ~ 11.7**	Increase rate by 1U/h	Increase rate by 1U/h	No change in rate
**7.8 ~ 10.0**	No change in rate	No change in rate	No change in rate
**6.1 ~ 7.8**	No change in rate	Decrease rate by 50%	Hold insulin infusion
**5.5 ~ 6.1**	• Hold insulin infusion • Check BG every 2 h • Restart infusion if BG > 10 mmol/L		
**3.9 ~ 5.5**	• Hold insulin infusion • Check BG every 30 min until BG > 5.5 mmol/L, resume BG checks every 2 h • Restart infusion at 1/2 the prior infusion rate if BG > 10 mmol/L		
**2.8 ~ 3.9**	• Give 25 mL D50 • Check BG every 30 min until BG ≥ 5.5 mmol/L, resume • BG checks every 2 h		
**≤ 2.8**	• Give 50 mL D50 • Check BG every 15 min until BG ≥ 3.9 mmol/L • When BG ≥ 3.9 mmol/L, check BG every 30 min until BG ≥ 5.5 mmol/L. Give 50 mL D50, followed by a continuous intravenous infusion of D10 • After BG ≥ 5.5 mmol/L, resume BG checks every 2 h • Restart infusion at 1/2 the prior infusion rate if BG > 10 mmol/L		

*BG* blood glucose, *mmol* millimoles, *L* liter, *U* units, *h* hour, *D50* 50% dextrose solution, *D10* 10% dextrose solution, *mL* milliliters

#### Anesthesia management

1. Preoperative: Informed consent will be signed preoperatively. Potential participants will be identified by reviewing the inpatient list or surgery schedule, and patients will be visited to sign the informed consent and baseline information will be collected. For patients included in CG group, a disposable glucose monitoring probe is placed and connected to the monitoring system 6 h prior to surgery.
2. Intraoperative: reliable intravenous access will be established after the patient enters the operating room, and electrocardiograph, blood pressure, pulse oximetry, end-expiratory carbon dioxide, and bispectral index (BIS) monitoring will be routinely performed. After adequate preoxygenation, standardized induction of anesthesia is started with propofol or etomidate, sufentanil or fentanyl, rocuronium bromide, or *cis*-atracurium. The actual drug dose should be adjusted by a qualified anesthesiologist based on the patient’s circulatory changes and surgical tolerance. Intraoperative anesthesia is maintained using total intravenous anesthesia, inhalation anesthesia, or a combination of static and inhalation anesthesia to maintain a stable depth of anesthesia. All medications should be recorded in detail and the patient should be transferred to the ward or recovery room or intensive care unit (ICU) for observation after the surgery.
3. Postoperative analgesia: After the surgery, patients routinely use a patient-controlled analgesic device with a recommended analgesic formulation of sufentanil 250 μg + ondansetron 20 mg + saline to 250 ml, with specific parameters of the analgesic pump adjusted according to the patient’s condition. With the regular postoperative analgesic dose, if the patient still has moderate or above postoperative pain (defined as: numerical rating scale ≥ 4/10), additional analgesic drugs can be added with the permission of the physician in charge, but the reason for administration and the type of drug administered should be recorded in detail.

#### Criteria for discontinuing or modifying allocated interventions {11b}

Interventions will be suspended in case of unintended or adverse events, or incomplete information collection that may has negative impact on the acquisition and interpretation of data, or in case of serious protocol deviations and implementation errors. Participating patients may withdraw from the study at any time and for any reason they wish, without consequence.

#### Strategies to improve adherence to interventions {11c}

For the participants in the intervention group, the alarm function of the CGM device will be turned on during LT. The device will continuously alert when the blood glucose value monitored by CGM is < 7.8 mmol/L or > 10.0 mmol/L, or the rate of change is > 1.67 mmol/(L∙min), in order to remind the investigator to reconfirm the blood glucose value using ABG test.

#### Relevant concomitant care permitted or prohibited during the trial {11d}

A conventional anesthetic management protocol is used intraoperatively. In the absence of clear indications, the control group should avoid two blood glucose monitoring intervals of less than 2 h. ABG testing in the intervention group should be performed only when the blood glucose value monitored by CGM is < 7.8 mmol/L or > 10.0 mmol/L, or the rate of change is > 1.67 mmol/(L- min).

#### Provisions for post-trial care {30}

No provisions or restrictions for post-trial care.

### Outcomes {12}

#### Primary outcomes

Intraoperative glucose coefficient of variation. In this study, the intraoperative coefficient of variation of blood glucose will be extracted from the CGM data for each participant (from the beginning of skin incision to the end of suture).

#### Secondary outcomes

This study intends to further assess the effectiveness of CGM-based intraoperative glucose management in LT by mean intraoperative glucose values, as well as rates of hypoglycemic events and hyperglycemic events. The accuracy of CGM for intraoperative glucose monitoring will be evaluated by overall clinical mean relative error and Clarke error grid analysis. The safety of CGM for intraoperative glucose monitoring will be evaluated by the incidence of severe adverse reactions (SAEs).

Additionally, this study intends to evaluate the prognostic improvement of patients with different glucose monitoring modalities by comparing the incidence of SSIs, other infections, postoperative liver function, and major adverse cardiovascular events (MACE) in the two groups at 3 months postoperatively.

### Participant timeline {13}

**Fig. 2:**
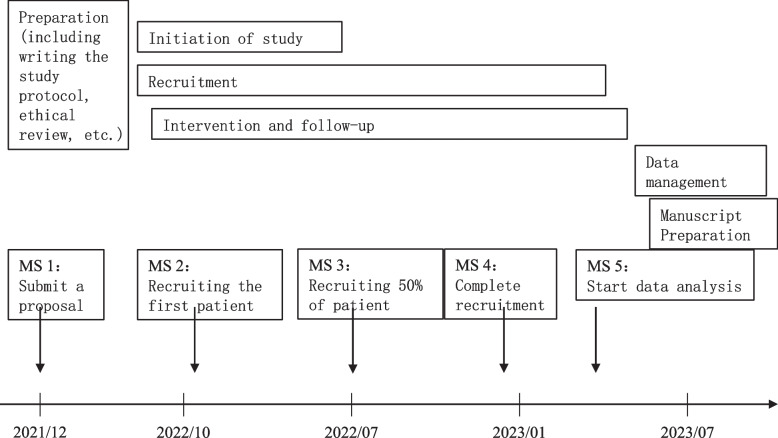
Study schedule

The study schedule is shown in Fig. 2.

### Sample size {14}

The intervention group is the continuous ambulatory glucose monitoring group (CG group), and the control group is the conventional arterial blood glucose monitoring group (C group). According to the previous literatures and our pre-experiment results, the intraoperative CV % in the liver transplant control group is about 40%, and it is expected that the intraoperative CV % in the intervention group could be reduced by 20%. Thus, we set bilateral α = 0.05 with 80% power, according to the sample size formula:

$$\begin{array}{cc}N=\frac{{\left({Z}_{a}+{Z}_{B}\right)}^{2}\left(1+\frac{1}{k}\right)P(1-P)}{{(Pe-{P}_{c})}^{2}}& p=\frac{Pe+kPc}{1+k}\end{array}$$

We estimate that the sample size for each group should be 78 cases. We set the sample size for each group to 90 to allow for up to 10% dropouts.

### Recruitment {15}

All patients proposed for orthotopic liver transplantation are screened for inclusion and exclusion criteria one day prior to surgery.

### Assignment of interventions: allocation

#### Sequence generation {16a}

An investigator (Pan Liu) who will not be involved in the patient recruitment and treatment process uses SAS 9.2 software (SAS Institute Inc., Cary, NC, USA) to generate a 0 s and 1 s random sequence (1:1), corresponding to sequence numbers from 1 to 180 for each sequence.

#### Concealment mechanism {16b}

Random numbers, serial numbers, and group numbers will be placed in opaque sealed envelopes. Each sealed envelope will be sequentially numbered and kept in a secure location until the study is completed. After participants have signed informed consent and passed the inclusion and exclusion criteria screening, the same investigator (Pan Liu) will open the envelopes with the same serial number in order of enrollment and inform the anesthesiologist of the treatment assignment. Participants and study stuff will not be blinded to the treatment assignment. Only follow-up stuff will be masked to treatment assignment.

#### Implementation {16c}

The study draws up the 0 s random sequence as the CG group and the 1 s random sequence as the C group. The assignment results will be stored in an electronic system until the study ended.

### Assignment of interventions: blinding

#### Who will be blinded {17a}

Only judicial assessors of outcomes and data analysts will be blinded to the group assignment, whereas care providers and patients are not blinded.

#### Procedure for unblinding if needed {17b}

Not applicable, care providers are not blinded to intervention assignment.

### Data collection and management

#### Plans for assessment and collection of outcomes {18a}

##### Screening period

The day before surgery, an initial screening will be performed by the participating investigator (Lei Cui) based on the surgery request form, and for each recipient who passes the initial screening, the investigator should fully inform him/her of the purpose, steps, potential benefits, and risks of this study. After obtaining the participant’s signed informed consent, further screening is performed according to the inclusion/exclusion criteria and the following information is collected:

1. Record of signing the informed consent
2. Signed informed consent form
3. Demographic data: age, sex, height, weight, and body mass index (BMI)
4. Medical history and related information: diagnosis (indications for liver transplantation), comorbidities, previous and current glycemic control, as well as history of medication, lifestyle interventions, smoking, alcohol consumption, food and drug allergies, and surgical anesthesia
5. Laboratory tests: preoperative HbA1C, liver function, kidney function, blood routine, and coagulation tests
6. Other examinations: electrocardiogram, ultrasonic cardiogram, carotid artery ultrasound, and abdominal CT
7. Donor information: age, height, weight, BMI, cause of death, liver biopsy, and virological findings

*Intervention period*

1. Randomized recording
2. Blood glucose: blood glucose, number of arterial blood gas checks, insulin and glucose administration (frequency and doses)
3. Surgery: length of surgery, surgical method, length of anhepatic phase, warm ischemia, and cold ischemia time
4. Anesthesia: duration of anesthesia, intraoperative drug use (drug name and dose used), blood pressure, cardiac output, stroke volume variation, body temperature, and BIS

##### Follow-up period

Patients were followed up on postoperative days 1 to 7 and days 15, 30, 60, and 90. Follow-up will be conducted by the investigator in the ICU or general ward during hospitalization and by telephone after discharge, respectively. The following information will be collected during the follow-up period:

1. Incidence of SSIs
2. Incidence of other infections: abdominal, pulmonary, and urinary tract infections
3. Postoperative liver function: liver function and coagulation tests on postoperative days 1–7
4. Incidence of MACE: cardiovascular death, myocardial infarction, and stroke
5. Length of postoperative ICU stay and hospitalization
6. All-cause mortality within 30 days
7. Re-hospitalization rate within 90 days
8. Secondary transplantation rate within 90 days

#### Plans to promote participant retention and complete follow-up {18b}

The investigator will explain to patients the importance and specific arrangements for completing follow-up visits on time and will encourage patients to complete them throughout the trial. Patients may withdraw from the trial at any time during the follow-up period without giving a reason. The investigator will perform complete-case analysis for all the outcomes. If more than 5% of the data are found to be missing for the primary outcome, a sensitivity analysis will be performed using multiple imputation.

#### Data management {19}

Data management for the study is performed by separate investigators for raw data management, case report form (CRF) design, data archiving, and storage management of the clinical and specimen information database.

CRF design and recording: we designed a CRF based on standard metadata. Investigators should record the original observations to the paper CRF in a timely, complete, and accurate manner.

Data archiving: The clinical information data of patients are systematically summarized and organized on an individual case basis with a timeline. After the CRFs are completed and signed by the investigator, two other investigators who are blinded to treatment assignment will use the EpiData 3.10 database system for data entry and management. All data files are transferred out to SPSS files at the end of the study and then statistically analyzed.

#### Confidentiality {27}

The electronic data in this study will be managed by dedicated personnel. The personal patient information will be appropriately hidden, only study-related data will be retained for statistical analysis. All CRF will be placed in a locked cabinet and managed by dedicated personnel. Researchers should follow professional confidentiality rules and must keep all personally identifiable information of patients as well as medical information confidential. At the end of the submission, paper CRFs are placed in a special locked cabinet and stored for 3 years before destruction, and electronic data are stored encrypted after hiding patients’ personal information. All work is assigned to a dedicated person.

#### Plans for collection, laboratory evaluation, and storage of biological specimens for genetic or molecular analysis in this trial/future use {33}

In this study, blood samples will be collected from patients for intraoperative arterial blood gas analysis, with the number of collections depending on the length of the procedure and changes in blood glucose. Patient blood is collected once a day for 7 days postoperatively for liver function and coagulation tests. Collected blood specimens will be disposed of at the end of the study.

### Statistical methods

#### Statistical methods for primary and secondary outcomes {20a}

Continuous variables such as CV%, mean blood glucose, and liver transaminases levels and other indicators will be tested for normality using Kolmogorov–Smirnov test. Measures satisfying normal distribution will be expressed as mean ± standard deviation, and median (interquartile range) when data are not normally distributed. Qualitative data such as the incidence of hypo/hypoglycemia, SSIs, and MACE will be expressed as frequencies (percentages).

Normally distributed data will be compared between two groups using *t*-test, multiple groups will be compared using ANOVA, and non-normally distributed data will be compared using Mann–Whitney *U*-test. The chi-square test will be used for comparison between groups for numerical data. Comparisons of CV%, the primary outcome, will be made using either the *t*-test or the Mann–Whitney *U* test.

Repeated measures will be compared using analysis of variance (ANOVA). Blood glucose analysis will be performed using multifactorial analysis.

In this study, the accuracy of CGM monitoring will be tested simultaneously for patients in the CG group. Pearson linear correlation analysis will be applied to determine the correlation between CGM- and ABG-derived glucose levels; Clarke error grid analysis will be used to evaluate the consistency between CGM- and ABG-derived glucose levels. The formula: [ABG glucose − CGM glucose)/ABG glucose] × 100% will be applied to calculate the overall clinical mean relative error. If this error value is ≤ 17% and the sum of the data falling in areas A and B in the Clark error grid analysis is not less than 95% of the total, the accuracy will be judged to be satisfactory.

All analyses will be performed using SPSS 26.0 (SPSS, USA) and STATA 14.0 (StataCorp LP, College Station, TX).

#### Interim analyses {21b}

A primary analysis will be conducted for this study and no interim analysis is planned.

#### Methods for additional analyses (e.g., subgroup analyses) {20b}

We will perform subgroup analysis based on the value of HbA1C (≤ 6.5% vs 6.5 ~ 8.5%). “*P*” value < 5% to be considered statistically significant. The differences of outcomes between subgroups will be evaluated using interactive subgroup analysis.

#### Methods in analysis to handle protocol non-adherence and any statistical methods to handle missing data {20c}

All randomized participants will be included in the main analyses. An intention-to-treat analysis will be carried out relating to any protocol non-adherence or dropouts. Per-protocol dataset will also be performed for any of the missing data. Besides, we anticipate little loss to follow-up for the primary outcome. Nevertheless, if there is more than 5% missing data for the primary outcome, we will perform a sensitivity analysis using multiple imputations for the missing data.

#### Plans to give access to the full protocol, participant-level data, and statistical code {31c}

The full protocol is publicly available. The public can obtain de-identified participant-level data and statistical code by reasonable written request to the principal investigator.

### Oversight and monitoring

#### Composition of the coordinating center and trial steering committee {5d}

Being a single-center study, the principal investigator (Yi Duan) and whole team are responsible for patient recruitment, data collection, maintaining the protocol, and patient follow-up. The Data Safety Monitoring Board (DSMB) will frequently monitor the study and report on trial quality control.

#### Composition of the data monitoring committee, its role and reporting structure {21a}

The DSMB is responsible for ensuring the safety of patients participating in the trial. The DSMB consists of three independent individuals, including statisticians and clinical decision makers. The DSMB meets at least once a year. The DSMB declares no competing interests.

#### Adverse event reporting and harms {22}

Treatment-related clinical adverse reactions including puncture site hematoma, infection, hypoglycemia, and hyperglycemia will be considered. The investigator should inform the participant of any emerging adverse events, and the participant should also report changes in condition truthfully in accordance with the protocol. The investigator should keep detailed records of all adverse events, including symptoms, time of presentation, duration, management measures, and effects, and evaluate their relevance to the study treatment. Investigators should be alert for serious adverse events (SAEs) such as severe hypoglycemia (< 3. 0 mmol/L), cardiac arrest due to dysglycemia, or death. Protecting the safety of the participants is the primary responsibility of the investigator. It should also be reported to the ethics committee, data monitoring committee, and relevant regulatory agencies, indicating the expectedness, severity, severity, and causality. DSMA regularly reviews study data and adherence to the protocol and makes recommendations regarding continuation, modification, or termination of the trial when necessary.

#### Frequency and plans for auditing trial conduct {23}

There is no interim audit for this study. After completion of the 180-case registry, a review will be conducted by a study supervisor independent of the study team. The Ethics Committee and DSMA have the right to review study data records and reports at any time, while maintaining confidentiality.

#### Plans for communicating important protocol amendments to relevant parties (e.g., trial participants, ethical committees) {25}

According to hospital regulations, the ethics committee should be notified of any protocol changes in the study with an application for approval. No further procedures allowed until approval.

#### Dissemination plans {31a}

The results of this trial will be published in a peer-reviewed journal.

## Discussion

This is a randomized, prospective, unblinded, study evaluating the potential superiority of CGM-based intraoperative glucose management. Low GV in clinical trials indicates improved postoperative outcomes and therefore was chosen as the primary endpoint. Secondary endpoints include mean intraoperative glucose levels and the incidence and extent of both hyperglycemic and hypoglycemic disorders. Furthermore, 3-month follow-up will help us explore whether intraoperative CGM monitoring could reduce the incidence of post-LT infectious events such as SSIs and MACEs and thus improve patients’ prognosis.

To the best of our knowledge, this is the first study investigating the prognostic role of CGM-based intraoperative glucose management in LT. The current study aims to realize continuous dynamic monitoring of intraoperative glucose and clarifies the effectiveness and feasibility of CGM for intraoperative glucose monitoring. Moreover, this study for the first time used GV coefficient as a key indicator of intraoperative glucose management and explored the impact of high GV on postoperative glucose-related complications, in addition to hyperglycemia and hypoglycemia.

This study has the following advantages. First, the study is conducted on liver transplant recipients. Most patients with end-stage liver disease have glucose metabolism disorders, and LT surgery is a traumatic procedure with high intraoperative doses of catecholamines, glucocorticoids, and other drugs that affect glucose metabolism, which leads to a higher incidence of intraoperative glucose disorders in LT recipients than in other procedures and often more severe. The high incidence and severity of glucose disorders help to verify the real-time effectiveness of novel glucose regulation model. Second, the study used arterial blood gas glucose as the gold standard and clinical overall mean relative error and Clarke error grid analysis to assess the accuracy of CGM for intraoperative glucose monitoring. The results of the study are therefore reliable and may be applicable to other major procedures. Finally, this study will be followed up for 3 months after surgery to fully reveal the impact of intraoperative GV and glucose levels on postoperative glucose-related complications.

This study has a few limitations. First, the study is not double-blinded, and the empirical increase in the number of arterial blood gas tests could have an impact on the effectiveness of glucose management. To avoid investigator bias, a detailed and objective monitoring protocol is developed in this study, and the investigators were adequately trained. Only investigators who passed the assessment are allowed to participate in this study. Second, this is a single-center study. In our liver transplantation center, the recipients are predominantly hepatitis B cirrhosis or alcoholic cirrhosis, and the procedure is primarily classical liver transplantation. Thus, further validation is warranted before applying these findings to patients with other types of end-stage liver disease or surgical modalities.

## Trial status

This study has been approved by the Ethics Committee of Beijing Tsinghua Changgung Hospital (approval number: 21288–0-02) and is registered at www.chictr.org.cn (ChiCTR2200055236). Patient recruitment for this study is expected to begin in October 2022 and completed by January 30, 2023.

## Data Availability

The investigators’ team has access to the dataset. Data can be provided on justified request with the approval of corresponding author.

## Abbreviations

SSIs: Surgical site infections
LT: Liver transplantation
GV: Glycemic variability
CV%: Coefficient of variation
CGM: Continuous glucose monitor
NMA: National Medical Products Administration
ABG: Arterial blood gas
MELD: Model for end-stage liver disease
BIS: Bispectral index
ICU: Intensive care unit
SAEs: Severe adverse reactions
MACE: Major adverse cardiovascular events
BMI: Body mass index
HbA1C: Glycosylated hemoglobin
SVV: Stroke volume variation
CRF: Case report form (CRF)
